# Susceptibility to the novel coronavirus disease (COVID-19) is associated with ABO and Rh blood groups: a case-control study from Afghanistan

**DOI:** 10.1186/s43042-020-00124-x

**Published:** 2021-01-05

**Authors:** Khyber Saify, Mohammad Sarwar Alborz, Mostafa Saadat

**Affiliations:** 1 Department of Biology, College of Education Sciences, Kunduz University, Kunduz, Afghanistan; 2 Department of Nutrition, College of Medical Sciences Kabul, Abu Ali Ibne-Sina Medical Science University, Kabul, Afghanistan; 3 Department of Biology, College of Sciences, Shiraz University, Shiraz, 71467-13565 Iran

**Keywords:** COVID-19, Susceptibility, ABO, Rh, Blood groups

## Abstract

**Background:**

There are preliminary studies about the association between COVID-19 and ABO phenotypes and the results are controversial. There are only two studies which investigated the association of Rh blood groups in addition to ABO with COVID-19; however, in the statistical analysis ABO and Rh blood groups have been considered separately. Therefore, the present case-control study was performed to determine the association of COVID-19 with ABO blood groups considering the Rh blood groups simultaneously. The study was conducted in Kunduz COVID-19 treatment specific center, Spin-Zar Hospital (Kunduz Province, North East Afghanistan). A total of 301 confirmed COVID-19 cases and 1039 healthy blood donors as control group were included in the study.

**Results:**

The Rh^−^ phenotype strongly increased the risk of COVID-19 (OR = 2.97, 95% CI 1.86–3.89, *P* < 0.001). Although blood group A increased the risk of developing COVID-19, the association did not reach statistical significance. In analysis of the combination phenotypes, the A^−^ blood group remarkably increased the risk of COVID-19 (OR = 7.24, 95% CI 3.62–14.4, *P* < 0.001). Multivariate analysis revealed that the interaction of Rh and ABO is significant (*P* < 0.013).

**Conclusion:**

These findings indicate that susceptibility to COVID-19 is strongly associated with A^−^ blood group.

## Background

Severe acute respiratory syndrome coronavirus 2 (SARS-CoV-2), causing coronavirus disease-2019 (COVID-19), is distributed to almost all countries in the world and until now (1 October 2020), more than 32,469,000 confirmed cases with 1,027,000 death have been reported from all around the world. On 24 February 2020, the first COVID-19 case was reported in Afghanistan in Herat Province and in a short time, it spread throughout the country [1]. As of 1 October 2020, there have been 39,285 COVID-19 cases with 1458 deaths in Afghanistan.

Susceptibility to COVID-19 is a multifactorial trait like other infectious disease. Several genetic elements and environmental factors are involved in its etiology. It should be noted that host and viral genetic variations might be involved in the pathogenesis of the disease [2–4]. There are several ecologic and case-control studies investigated the association between common genetic polymorphisms and susceptibility to COVID-19, as well as its mortality [5–13].

The association of genetic polymorphism in blood groups with susceptibility to some infectious diseases has been well established [14]. Very recently, it has been reported that the prevalence and mortality of COVID-19 is associated with ABO (MIM: 616093) and Rh (MIM: 111680) blood groups, based on an ecologic study. Countries with higher A phenotype and Rh negative blood group showed higher values of the prevalence and mortality rate [5]. However, studies on the association between COVID-19 and ABO phenotypes are at preliminary stage and the results are controversial [15–28].

There are three studies which investigated the association of Rh blood groups in addition to ABO with COVID-19; however, in the statistical analysis, ABO and Rh have been considered separately [15, 23, 25]. Therefore, the present case-control study was performed to determine the association of COVID-19 with ABO blood groups considering the Rh blood groups simultaneously.

## Methods

### Participants

This case-control study was accomplished during August and September 2020 in Kunduz COVID-19 treatment specific center, Spin-Zar Hospital supported by Ministry of Public Health of Afghanistan and JACK organization (Kunduz Province, North East Afghanistan). Afghanistan has a heterogeneous population [29–32]; therefore, we selected the participants (patients and controls) from Muslims living in Kunduz City.

A total of 301 confirmed cases of COVID-19 patients were included in the study. From these 136, 127, and 38 individuals were Pashtuns, Tajiks, and Uzbeks, respectively. A control sample of 1039 healthy blood donors was also included in the study. From these 486, 424, and 126 individuals were Pashtuns, Tajiks, and Uzbeks, respectively. In order to increase the statistical power of the analysis, the data were pooled.

The present study was performed in accordance with the ethical codes of the Declaration of Helsinki and was approved by the ethics committee of our university. The information for case group was obtained by a questionnaire. Oral informed consent was obtained from each participant before the study.

### Measurements

Infection by Covid-19 was confirmed using real-time polymerase chain reaction (RT-PCR) test in the Kunduz COVID-19 treatment specific center, Spin-Zar Hospital. The sample test procedure is as follows setups. At first, nasopharyngeal swabs were collected by Citoswab collection and transport kit (Citotest Labware Manufacturing Co., Ltd.). Total RNA was extracted from nasopharyngeal samples by QIAamp Viral RNA Mini Kit (Cat No. 52906 QIAGEN) according to the manufacturer’s instructions. RNA samples were used for RT-PCR by detection Kit DA0931, for 2019 Novel Coronavirus (2019-nCoV) RNA (PCR-Fluorescence Probing) Da An Gene Co., Ltd. of Sun Yat-sen University of China, using an Applied Biosystems 7500 Real Time PCR System. The reaction mixture was subjected to initial denaturation at 95 °C for 15 min, followed by 45 cycles of 95 °C for 15 s, 55 °C for 45 s, and 72 °C for 40 s set. The case samples compared with the positive and negative C_T_ curve according to the manufacturer’s instructions.

Blood samples were collected without anti-coagulant by finger prick with sterile lancet and the ABO and Rh blood group were determined based on standard method of antigen-antibody agglutination test using anti-sera (anti-A, anti-B, and anti-D) kit (CinnaClone II Ltd., Iran).

### Statistical analysis

For the control group, the observed frequencies of the ABO phenotypes were assessed for Hardy-Weinberg equilibrium (HWE) using the *χ*^2^ statistic [33].

Our approach for analysis of the data consisted of three steps. At first, the frequencies of blood group O (for ABO blood groups) and Rh^+^ (for Rh blood groups) compared separately between cases and controls, using logistic regression analysis. In the logistic model, the blood group O (for ABO blood groups) and Rh^+^ (for Rh blood groups) were considered as reference categories and odds ratios (ORs) and their 95% confidence intervals (95% CI) of COVID-19 risk were estimated. The genes encoding the ABO and Rh blood groups are located on human chromosomes 1 and 9, respectively [34, 35]. It means that they are independent loci. In the second step, combination phenotypes of ABO and Rh blood groups compared between cases and controls. In the analysis, O+ phenotype was used as reference group. Finally, multivariable logistic regression analysis was used to investigate the interaction of ABO and Rh phenotypes at the third step. Statistical analysis was performed using the SPSS software (SPSS Inc., Chicago, IL, USA; version 11.5). A *P* value less than 0.05 was considered as statistically significant.

Sample size which is required for identification a significant difference in phenotypic frequency analysis for an effect size of 0.15 (small-medium effect) with assumptions of α = 0.05, β = 0.20, Lambda = 14.88, and *df* = 3 were calculated using the GPOWER (www.psycho.uni-duesseldorf.de/aap/projects/gpower) software (version 3.1.3). A minimum sample of 630 would be sufficient. The present case-control study is more than sufficiently powered with an *n = 1337* to detect a small-medium effect for comparing the frequency of blood groups between the two groups.

## Results

There was no significant difference between COVID-19 cases and healthy controls for ethnicity of the participants (*χ*^2^ = 0.28, df = 2, *P* = 0.869). Therefore, the ethnic groups were pooled. Table 1 shows the frequency distribution of ABO and Rh blood groups in COVID-19 patients and control group. The allelic frequencies of A, B, and O in control group were 0.2233, 0.1707, and 0.6060, respectively. The expected values of the ABO phenotypes based on HWE showed high similarity with the observed frequencies (*χ*^2^ = 0.67, *df* = 1, *P* = 0.411). The Rh^−^ blood group compared to the Rh^+^ phenotype strongly increased the risk of COVID-19 (OR = 2.97, 95% CI 1.86–3.89, *P* < 0.001). Although the A phenotype and the A + AB blood groups versus to the O phenotype increased the risk of COVID-19, the association did not reach statistical significance (Table 1).

**Table 1:** Frequency of ABO and Rh blood groups in control and COVID-19 patient groups

Polymorphisms	Controls (%)	COVID-19 (%)	OR	95% CI	***P***	OR^**a**^	95% CI	***P***
**ABO**								
O	377 (36.39)	98 (32.56)	1.0	–	–	1.0	–	–
A	330 (31.85)	112 (37.21)	1.30	0.95–1.77	0.090	1.31	0.96–1.79	0.086
B	243 (23.46)	61 (20.26)	0.96	0.67–1.38	0.848	0.97	0.68–1.40	0.910
AB	86 (8.30)	30 (9.97)	1.34	0.83–2.15	0.221	1.36	0.84–2.19	0.204
A + AB	416 (40.15)	142 (47.18)	1.31	0.98–1.76	0.068	1.32	0.95–1.77	0.063
**Rh**								
Rh^+^	955 (92.18)	245 (81.40)	1.0	–	–	1.0	–	–
Rh^−^	81 (7.82)	56 (18.60)	2.97	1.86–3.89	< 0.001	2.69	1.85–3.91	< 0.001
**Total**	1036 (100.0)	301 (100.0)						

^a^Adjusted for gender and ethnicity

In further analysis, the associations between combination of the ABO and Rh phenotypes and susceptibility to COVID-19 were investigated. The results were summarized in Table 2. The A^−^ vs O^+^ blood group remarkably increased the risk of COVID-19 (OR = 7.24, 95% CI 3.62–14.4, *P* < 0.001). The O^−^ *vs* O^+^ phenotype significantly associated with the risk of COVID-19, which is quite similar with the association between Rh^−^ and susceptibility to COVID-19 (Tables 1 and 2). It seems that Rh and ABO blood groups may have interaction with each other. To investigate this point, multivariable logistic regression analysis was done, using ABO and Rh phenotypes as independent variables. Analysis revealed a significant interaction of Rh and ABO blood groups (*P* < 0.024). The same results were obtained after adjustment for ethnicity and gender of the participants (Tables 1 and 2).

**Table 2:** Combination phenotypes of the ABO and Rh blood groups and susceptibility to COVID-19

Polymorphisms		Controls (%)	COVID-19 (%)	OR	95% CI	***P***	OR^**a**^	95% CI	***P***
**ABO**	**Rh**								
O	+	344 (33.20)	76 (25.25)	1.0	–	–	1.0	–	–
A	+	315 (30.41)	88 (29.24)	1.26	0.89–1.78	0.180	1.27	0.90–1.80	0.167
B	+	213 (20.56)	53 (17.61)	1.12	0.76–1.66	0.550	1.12	0.75–1.66	0.559
AB	+	83 (8.01)	28 (9.30)	1.52	0.93–2.50	0.094	1.54	0.93–2.54	0.088
O	−	33 (3.19)	22 (7.31)	3.01	1.66–5.46	< 0.001	2.97	1.63–5.43	< 0.001
A	−	15 (1.45)	24 (7.97)	7.24	3.62–14.4	< 0.001	6.95	3.45–13.9	< 0.001
B	−	30 (2.89)	8 (2.66)	1.20	0.53–2.73	0.652	1.29	0.56–2.96	0.539
AB	−	3 (0.29)	2 (0.66)	3.01	0.49–18.3	0.231	2.88	0.46–17.9	0.257
**Total**		1036 (100.0)	301 (100.0)						

^a^Adjusted for gender and ethnicity

## Discussion

There are several studies reporting the relationship of ABO and Rh blood groups with susceptibility to COVID-19 or with outcome of the COVID-19 [15–28]. The results are inconsistent. However, it seems that the risk of COVID-19 is positively associated with A blood group compared with O phenotype [17, 18, 24, 26, 28]. Our present finding, confirmed the relationship of ABO genetic system with susceptibility to COVID-19. Very recently, a genome-wide scan study indicated that human chromosome segment 9q34.2 is associated with severe form of COVID-19 with respiratory failure [36]. It should be mentioned that the ABO locus is located on the 9q34.2 [34]. Taken together, these findings indicate that this locus might be involved in the etiology of the disease.

The novelty of the present study is the strong positive association between Rh^−^ blood group and the risk of developing COVID-19. This finding is consistent with the result of a recently published ecologic study. Based on data, from 86 countries, the authors reported that frequency of the Rh^−^ blood group is positively associated with the prevalence, mortality, and case-fatality of COVID-19. It means that prevalence of COVID-19 is higher in countries where the Rh^−^ blood group is more frequent [5].

COVID-19 is an infectious and communicable disease. Therefore, several factors are involved in the pathogenesis of the disease. In order to confirm the role of the Rh blood groups on susceptibility to COVID-19, replication of this study in other countries is recommended with respect to these factors.

## Conclusions

The present case-control study indicated that there is a strong positive association between Rh^-^ blood group and the risk of COVID-19. Also, the present study revealed that among the combination phenotypes, the A^−^ blood group is significantly associated with the susceptibility to COVID-19.

## Supplementary Information

**Additional file 1.**

## Data Availability

All data generated or analyzed during this study are included in this published article and its supplementary information file.

## Abbreviations

CI: Confidence interval
COVID-19: Coronavirus disease-2019
HWE: Hardy-Weinberg equilibrium
OR: Odds ratio
PCR: Polymerase chain reaction
SARS-CoV-2: Severe Acute Respiratory Syndrome Coronavirus 2
